# Main social vulnerability indicators in the COVID-19 pandemic in Iran

**DOI:** 10.3389/fpubh.2023.1080137

**Published:** 2023-03-27

**Authors:** Shandiz Moslehi, Alireza Dehdashti, Behrad Pourmohammdi, Farin Fatemi

**Affiliations:** ^1^Health Management and Economics Research Center, Health Management Research Institute, Iran University of Medical Sciences, Tehran, Iran; ^2^Department of Health in Disasters and Emergencies, School of Health Management and Information Sciences, Iran University of Medical Sciences, Tehran, Iran; ^3^Social Determinants of Health Research Center, Semnan University of Medical Sciences, Semnan, Iran; ^4^Research Center of Health Sciences and Technologies, Faculty of Health, Semnan University of Medical Sciences, Semnan, Iran; ^5^Department of Parasitology, School of Medicine, Semnan University of Medical Sciences, Semnan, Iran

**Keywords:** social vulnerability, COVID-19, pandemic, indicators, Iran

## Abstract

**Background:**

Social factors play the main role in the vulnerability of exposed countries to disasters. The COVID-19 pandemic as a disaster is not an exception to this fact. This study aimed to determine the main social vulnerability indicators in the COVID-19 pandemic in Iran.

**Methods:**

This study was conducted during the period of 2021–2022 in three phases, including a systematic review, a virtual panel expert, and the Analytical Hierarchy Process. First, the draft of social vulnerability indicators in COVID-19 was extracted through a systematic review. Then, the extracted indicators were finalized and prioritized by the expert panel and the AHP, respectively.

**Results:**

Initially, the literature review found five domains and 38 indicators of social vulnerability in COVID-19. The outcome of the expert panel increased the related domains to six but decreased the indicators to 31. The three prioritized social vulnerability indicators that were determined by the AHP were population density, accessibility to healthcare facilities, and relevant services and vulnerable groups.

**Conclusion:**

Measuring social vulnerability with the identified indicators is valuable for addressing high COVID-19 incidence among socially vulnerable hotspot areas. Regarding the result of this study, further research should be conducted to validate the identified indicators.

## 1. Introduction

Social vulnerability plays the main role in exposing communities and countries to disasters and emergencies, even more so than other forms of vulnerability such as physical, economic, or environmental (1). Scholars have used a variety of terms to describe social vulnerability and also have determined the relevant indicators, especially in natural disasters. Cutter, as one of the first scientists in this field, described the social vulnerability concept as social factors that influence or shape the susceptibility of various groups to harm and that also govern their ability to respond (2–4). Several years later, Flanagan et al. developed the hierarchical social vulnerability index for the U.S. Center for Disease Control (CDC). He used indicators such as housing and transportation, minority status, household composition, and socioeconomic status for measuring the Social Vulnerability Index (SoVI) in disaster management in 2011 and updated this index in 2018 (5, 6). The SoVI is a tool that uses census data to identify and map places where a community may have more difficulty preventing human suffering and financial loss in a disaster. This tool is important for responding to incidents and disasters in an equitable way (7–9). Additionally, the literature review indicates that the communities with higher social vulnerability have more adverse outcomes during and following a disaster (10–12). Iran is a disaster-prone country, and due to its geographical and ethnic diversities, we have few studies and inadequate information about indicators of social vulnerability in Iran (1, 13, 14).

The incidence of COVID-19 in 2020 and its conversion to a pandemic worldwide in a short time confirmed the importance of social vulnerability among societies and countries again (15, 16). Iran, like many other countries, has been severely affected by the coronavirus pandemic (17). The pandemic caused casualties as well as great and irreparable economic and social damage to society, and the healthcare system was overwhelmed during the peaks of the pandemic (18–20). As social vulnerability is one of the effective factors in disaster impacts, this point had been generalized to the COVID-19 pandemic as a disaster in societies and countries (21–23). Therefore, one of the research priorities in Iran should be to identify the indicators of social vulnerability regarding COVID-19 and find strategies to decrease the social vulnerability to this tragic pandemic.

This study aimed to develop and prioritize the indicators of social vulnerability against the COVID-19 pandemic by applying the Analytical Hierarchy Process (AHP) approach in Iran.

## 2. Methods

This research was conducted in three stages in 2021–2022, as follows: First, a literature review to identify the social vulnerability indicators; second, an expert panel and consultation with experts to assess the feasibility of using the retrieved indicators due to the data accessibility and the context of Iran; and finally, using AHP to prioritize and give weight to the final main indicators.

### 2.1. Review of literature

A literature review was performed to review available published articles, documents, and reports. First, the research question and search strategy were developed. Then, to determine the nature and format of the main social vulnerability indicators in the COVID-19 pandemic, available evidence, including articles, documents, and reports, was searched and reviewed. This literature review was performed in partnership with a research team and medical information specialists through December 2020. The Web of Science, PubMed, and Google Scholar were searched for relevant articles. For keywords, the combination of the following Medical Subject Headings (MeSH) in consultation with our research team and experts, including an experienced medical information specialist, was used: “COVID-19,” “social vulnerability”, “management”, “indicator”, “epidemic”, “pandemic”, “outbreak”, and “infectious disease”. In addition, we searched specialized databases and websites such as the World Health Organization (WHO), the Federal Emergency Management Agency (FEMA), and the Michigan Department of Health and Human Services (MDHHS) with the mentioned keywords. Then, references and bibliographies of all relevant articles were reviewed to identify additional studies. All found studies and literature were entered into the EndNote software version X7.

Articles were selected by applying the inclusion and exclusion criteria. The inclusion criteria were as follows: (1) addressing the social vulnerability indicators and infectious diseases and (2) the availability of the full text of articles for free. The exclusion criteria were as follows: (1) articles published in non-English languages; (2) studies that solely focused on concepts or theoretical frameworks and did not provide metrics or measurement indicators; and (3) news articles, abstracts, and those studies for which full texts were not available.

Duplicate articles were eliminated *via* the EndNote software. Articles were screened by assessing the titles and abstracts for eligibility by two reviewers independently. The full text of the retrieved articles was reviewed by the research team. After screening studies based on the inclusion criteria, the final set of studies was summarized using a data extraction form. Data were charted and presented according to the research objective. The process of doing this phase with more details had been published earlier by one of the members of the research team in the present study (24).

### 2.2. Expert panel and consultation with experts

Expert panels in two rounds were planned from 15 January 2020 to 20 February 2021 to discuss the findings and examine the feasibility of the recommended indicators in Iran. Due to the conditions of COVID-19, a face-to-face panel was not possible, and telephone, mobile social media applications (Skype), and email were used to provide the experts' opinions. For this purpose, academics, policymakers, infectious disease specialists, and social and welfare experts in Iran were invited to develop discussions about the indicators of social vulnerability during the COVID-19 pandemic based on the results of the literature review.

A list of participants was prepared. After obtaining their permission and approval for the dates and time of the panel, an official invitation letter along with a research brief was sent to them by email or Skype. However, before participation, they were informed of the study's objective and considered a topic in the expert panel session. A discussion guide, due to the literature review (social vulnerability indicators in the COVID-19 pandemic), was developed to assess the feasibility of using the retrieved indicators in the data accessibility and context of Iran. Two members of the research team served as facilitators, presenting the questions most appropriate according to the participants' backgrounds and in relation to the study objective. Additionally, the participation was voluntary, and the participants could withdraw from the study without any consequence. The facilitators used an informed consent form for the participants and ensured that they felt free to answer any question, had the possibility of ending their participation in this study, and had the right to prevent or stop the recording. In total, eight experts participated in the study in addition to the research team. Two meeting sessions were coordinated *via* Skype for each round of the expert panel. Additionally, the comments of two experts were collected *via* email in each round of the expert panel. The Skype sessions were recorded.

Finally, the consensus between the opinions of the experts was reached by the facilitator of the meeting in such a way that at least 70% of the members agreed with including the indicators in the study (25). The indicators of social vulnerability in the COVID-19 pandemic in Iran that received less agreement than 70% were eliminated.

### 2.3. Prioritizing indicators using AHP

After finalizing the main social vulnerability indicators in the COVID-19 pandemic in Iran, a questionnaire was designed based on a pairwise comparison of all indicators, and finally, we estimated relative weights for selected indicators using the AHP technique.

The university professors who are specialists in the fields of disaster and emergency health, infectious disease, and social and welfare were asked to prioritize the importance of each of these indicators. To assess the reliability of the results, the consistency ratio (CR) was evaluated (26). In this stage of the study, 12 experts participated and estimated relative weights. The participants had to choose an indicator in the pairwise comparison questionnaire and identify the degree of more or less importance of each selected indicator. To help with the comparison, a nine-point scale of importance was created (27). The suggested numbers were used to express the degree of importance between each of the two indicators based on the nine-point scale as shown in Table 1. Then, the researchers calculated the weights for each indicator.

**Table 1:** The nine-point scale and description of its items.

**Definition**	**Degree of importance**
Equally importance	1
Moderately importance	3
Strongly importance	5
Very Strongly importance	7
Extremely importance	9

## 3. Results

A total of eight and twelve experts with years of experience in disasters and emergencies, infectious disease, and social and welfare participated in the expert panel and AHP phases of this study, respectively. The mean age of the participants was 40.3 ± 4.7, and their mean work experience was 12.1 ± 5.5 years.

### 3.1. Review of literature

The most repeated domains of social vulnerability to COVID-19 in studies were family composition and disability, public health infrastructures, racial and ethnic minorities, socioeconomic status, housing status, and transportation. Additionally, the relevant indicators for the mentioned domains, such as the percentage of single-parent households, the immigrant population, population density, unemployed rate, percentage of healthcare service workers, and percentage of the population who had access to health insurance, were extracted from the literature review. Then, they were adjusted as a draft of social vulnerability, including five domains and 38 indicators for the next stage.

### 3.2. Expert panel and consultation with experts

The expert panel was conducted in two rounds subsequently. For each round, two sessions were organized *via* Skype. During these two rounds, the experts discussed and reached a consensus agreement on domains and indicators that would be useful as a basis for developing a tool for assessing social vulnerability in COVID-19 in Iran. Additionally, the collected comments of two experts *via* email were involved in the including or excluding of domains and indicators in two rounds of an expert panel.

In the first round of the expert panel, the number of extracted domains from the literature review increased from five to six, according to the consensus of experts. In the draft version of domains from the literature review, education was accounted for as one of the indicators of the “household composition and disability” domain. The experts reached an agreement to categorize education as an individual domain of social vulnerability in COVID-19. Moreover, the five indicators that received <70% agreement were eliminated. The eliminated indicators in the first round were “no high school diploma” and “percentage of households with heads aged 60 years or older” from the socioeconomic domain, “income” and “percentage of children under poverty” from the socioeconomic status domain, and “population does not have a safe home to shelter in place” from the housing and transportation domain. Then, the following two indicators were defined for the education domain: “no high school diploma” and “percentage of the population with university education.” In total, there were 35 indicators at the end of the first round of the expert panel.

The second round of the expert panel was held by the same participants as the previous round. The domains and indicators were discussed again, and the number of domains remained unchanged but the number of indicators decreased from 35 to 31. The eliminated indicators included “percentage of households with children aging from 0 to 4 years” from the household composition and disability domain, “percentage of children with no access to the internet” from the socioeconomic status domain, “working population spend more than 60 min on commute” and “percentage of pedestrian and bike commuters” from the housing and transportation domain, and “hospital bed per capita” from the public health infrastructure domain. Additionally, the indicator of “place of living (urban or rural areas)” was added to the housing and transportation domain. Consequently, the final version of domains and indicators of social vulnerability in COVID-19 included 6 and 31 items, respectively (Table 2).

**Table 2:** Finalized domains and relevant indicators of social vulnerability in COVID-19.

**Domain**	**Indicator**
Household composition and disability	Aged 65 years or older
	Aged 17 years or younger
	Sex ratio
	Older than age 5 with a disability
	Single parent household
	Average number of family members
Race and minority status	Minority
	Immigrant population
Socioeconomic status	Percentage Living Below Poverty Line
	Gini coefficient
	Unemployment rate
	Proportion in at risk industries
	Proportion in at risk occupations
	Median household income
	Food security
Housing and transportation	Place of living (urban or rural area)
	Percentage of housing in structures with 10 or more units
	Percentage of mobile homes
	Percentage of persons in institutionalized group quarters such as prisons, nursing homes (already described), dormitories or schools
	Population density or crowding
	Percentage of households with no vehicle available
	Working population access to public transportation
	Means of essential transport during a crisis
Public health infrastructures	Percentage of healthcare service workers
	Reserved medical stocks
	Access to health insurance
	Number of healthcare facilities in the area
	Ratio between the number of hospitals to the number of population
	Percentage of households without access to safe drinking water
Education	No high school diploma
	The percentage of population with university education

### 3.3. Prioritizing indicators using AHP

The indicators were assessed due to the availability of data during the COVID-19 pandemic in Iran. Finally, 10 main indicators were selected. By applying the AHP method during the collective discussion, a group of twelve experts defined the importance of each indicator of social vulnerability in the COVID-19 pandemic. For determining the importance, the experts completed the pairwise comparison matrix by comparing all indicators in pairs.

**Figure 1:**
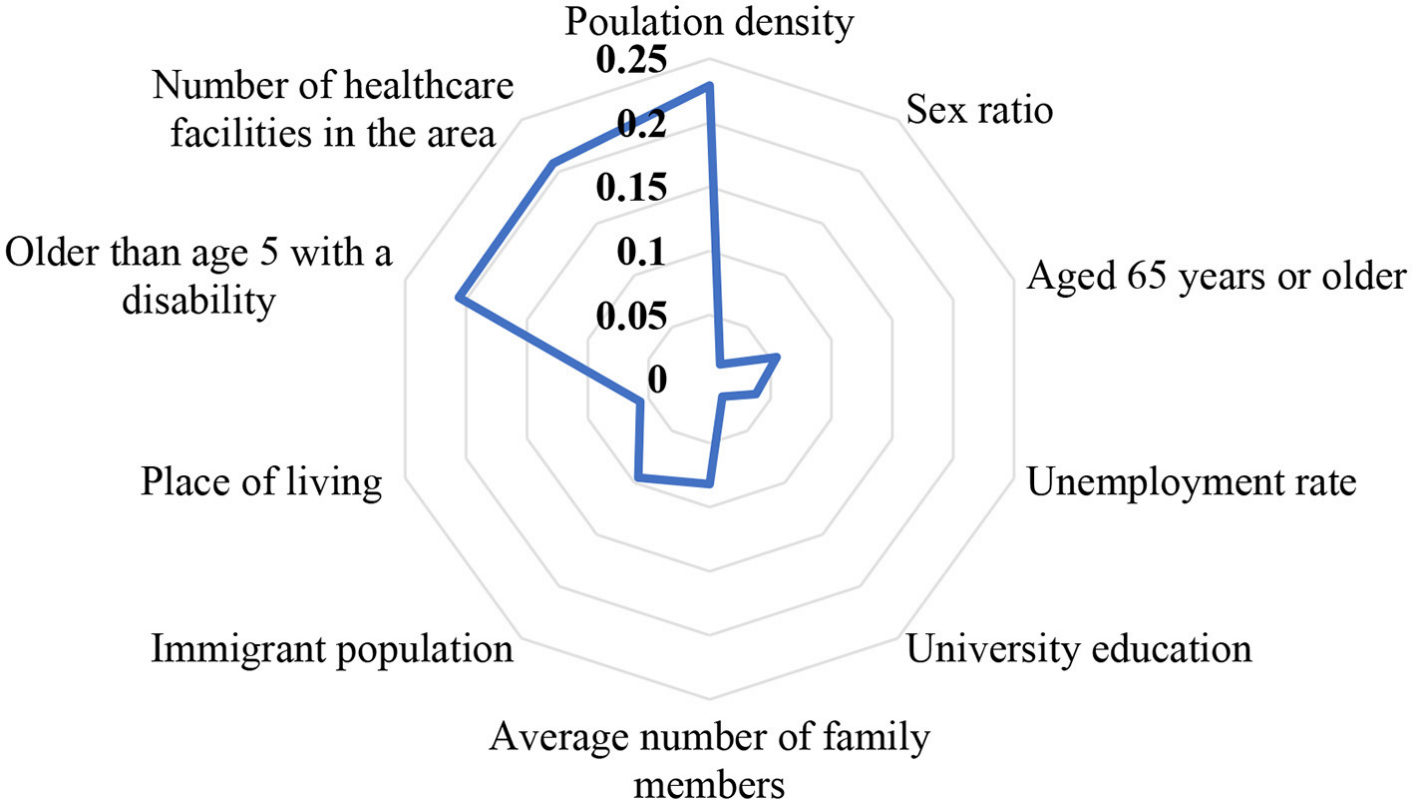
Radiated diagram of social vulnerability indicators in the COVID-19 pandemic in Iran.

The results of the pairwise comparison matrix and relative importance vector are presented in Table 3. According to Figure 1, the most important indicator is population density (ωC1 = 0.229); the second-most important indicator is access to healthcare facilities and relevant services (ωC10 = 0.208); vulnerable group is also the third-most important indicator (ωC9 = 0.206). Further, gender is the least important indicator (ωC2 = 0.014). The sum of the relative importance vectors is equal to 1. The consistency of the pairwise comparison was checked through face-to-face interviews with experts. Next, the consistency ratio was calculated to check the consistency of the judgments. The consistency index was (CI = 0.129), the consistency ratio was (CR = 0.087), which was lower than 0.1. This demonstrates that the pairwise comparison matrix is consistent.

**Table 3:** Group decision matrix and relative importance (priority) vector.

**Indicator**	**C1**	**C2**	**C3**	**C4**	**C5**	**C6**	**C7**	**C8**	**C9**	**C10**	**Relative importance (priority) vector**
C1	1.000	8.045	7.042	5.180	5.200	1.874	3.686	3.698	2.226	2.182	0.229
C2	0.124	1.000	0.171	0.150	0.887	0.131	0.136	0.161	0.126	0.118	0.014
C3	0.142	5.832	1.000	2.451	5.337	0.373	0.254	1.308	0.225	0.383	0.055
C4	0.193	6.681	0.408	1.000	2.874	0.323	0.295	0.938	0.171	0.143	0.038
C5	0.192	1.127	0.187	0.348	1.000	0.153	0.143	0.170	0.123	0.120	0.017
C6	0.534	7.622	2.682	3.093	6.520	1.000	0.352	1.345	0.212	0.315	0.082
C7	0.271	7.346	3.939	3.394	7.001	2.841	1.000	1.190	0.194	0.233	0.095
C8	0.270	6.210	0.765	1.066	5.890	0.744	0.841	1.000	0.173	0.183	0.057
C9	0.449	7.944	4.441	5.850	8.132	4.719	5.152	5.770	1.000	0.610	0.206
C10	0.458	8.485	2.610	7.007	8.310	3.175	4.283	5.462	1.639	1.000	0.208

C1, population density or crowding; C2, sex ratio; C3, aged 65 years or older; C4, unemployment rate; C5, university education; C6, average number of family members; C7, immigrant population; C8, place of living; C9, older than age 5 with a disability; C10, number of healthcare facilities in the area.

## 4. Discussion

This study aimed to develop the main domains and indicators of social vulnerability in the COVID-19 pandemic. Across the world, there have been many studies on the assessment and measurement of social vulnerability but most of them focused on natural disasters such as earthquakes or floods. Although these studies yielded significant insights into the conceptual meaning of social vulnerability, only a few of them included valid indicators (5, 7, 28–31). A few social vulnerability investigations have been conducted individually in Iran, but most of the studies in the world collected and weighed the relevant social vulnerability indicators in four broad categories, including population, housing, socioeconomic status, and physical distance (13, 24, 32). The literature review shows that epidemics are also affected by social vulnerability indicators such as housing, economics, transportation, and the environment in different communities (33, 34) and COVID-19 is no exception. One study in the United States showed that greater social vulnerability has a direct effect on the increased risk of COVID-19 infection and mortality (35).

Based on the results of this study, the main social vulnerability domain and indicators were housing and population density, respectively. Previous findings from the studies of vulnerability-relevant factors for COVID-19 have also confirmed our results. Living in high-density populations causes the spread of COVID-19 due to crowded areas and high physical contact with other people (36–38). Furthermore, vulnerable groups (*aged 65 years or older, aged 17 years or younger, older than age 5 with a disability, and single-parent households)* and public health infrastructure (*number of healthcare facilities in the area*) were the other considerable domains and indicators with the highest weight. These findings were confirmed by some worldwide studies during COVID-19. For instance, the results of two studies in Iran and the United States showed that children and the elderly are at higher risk of COVID-19 infection and related mortality and more vulnerable to receiving various medical, financial, and emotional needs during COVID-19 and previous epidemics (39, 40). Additionally, the literature review indicated that people with disabilities need support from rehabilitation centers and relevant service organizations to receive healthcare related to COVID-19 infection (41, 42). The results of one study showed that discrimination in access to healthcare facilities and services may lead to a higher prevalence of COVID-19 and the continuity of the transmission chain of the pandemic (39). Furthermore, one study in Brazil found that the great heterogeneity in hospital capacity across the country posed an important challenge for resource allocation to patients during the pandemic (43).

Since scientific data and viewpoints of experts were considered in the development of the main social vulnerability indicators in COVID-19, remote sensing methods and GIS are the appropriate tools for mapping social vulnerability in the COVID-19 pandemic in the case of existing data related to social vulnerability indicators (1, 16, 44). The output of this study, in the form of a questionnaire, is hoped to be used by most disaster scholars and practitioners in this field to improve the recognition of more vulnerable areas and prioritize the distribution of resources to ensure equity for residents during COVID-19 and other probable epidemics in the future.

There are some limitations to this study. First, only English articles, documents, and books were used in the literature review stage, and some important published training materials in other languages must have been missed. Second, due to social distancing principles in the COVID-19 pandemic, holding the expert panel sessions and AHP *via* social media or email was a time-consuming process. Data saturation was not also obtained, unlike in the physical environment of expert panel sessions, where the experts express their viewpoints face-to-face. Furthermore, more focus was on the indicators of social vulnerability in this study because quantitative data were available from the latest census in Iranian databases.

## 5. Conclusion

The main social vulnerability indicators in COVID-19 were determined in Iran during this study. The previous studies showed that regions with higher social vulnerability experienced greater mortality rates during the COVID-19 pandemic. Thus, we determined the main social vulnerability in the COVID-19 pandemic in this study. In the future, we need to measure the social vulnerability index in different regions of Iran and compare the results with other parts of the world that have done similar social vulnerability studies. In the next stage, public health actions will be predicted for the regions that were socially vulnerable to becoming COVID-19 hotspots in Iran. Additionally, recognizing the social vulnerable regions from COVID-19 helps to prepare the multiagency response teams and required measures for future epidemics or pandemics management.

However, there is a need to conduct empirical studies on the validity and generalizability of identified indicators in the COVID-19 pandemic in different regions of Iran. Although collecting data about considered indicators would be difficult because of inadequate secondary data, there may be a need to decrease the number of developed indicators in this study.

## Data availability statement

The datasets presented in this study can be found in online repositories. The names of the repository/repositories and accession number(s) can be found in the article/Supplementary material.

## Ethics statement

This study was approved by the Ethics Committee Review Board at Semnan University of Medical Sciences (IR.SEMUMS.REC.1399.110). All the participants signed a consent form and were informed on the purpose of the study prior to interview as per local protocol on research ethics.

## Author contributions

FF and BP collected data and contributed to entering data into the dataset. FF, SM, and AD designed the study, analyzed data, and prepared the manuscript. All authors read and approved the final manuscript.
